# Dermatological Manifestations in COVID-19: A Case Study of SARS-CoV-2 Infection in a Genetic Thrombophilic Patient with Mthfr Mutation

**DOI:** 10.3390/pathogens12030438

**Published:** 2023-03-10

**Authors:** Gabriela Gomes Celestino, Marla Karine Amarante, Eliana Carolina Vespero, Eliandro Reis Tavares, Lucy Megumi Yamauchi, Érika Donizetti Candido, Danielle Bruna Leal de Oliveira, Edison Luiz Durigon, Sueli Fumie Yamada-Ogatta, Ligia Carla Faccin-Galhardi

**Affiliations:** 1 Departamento de Microbiologia, Universidade Estadual de Londrina, Londrina 86057-970, Paraná, Brazil; 2 Departamento de Patologia, Universidade Estadual de Londrina, Análises Clínicas e Toxicológicas, Londrina 86038-350, Paraná, Brazil; 3 Laboratório de Virologia Clínica e Molecular, Universidade de São Paulo, São Paulo 05508-000, São Paulo, Brazil; 4 Hospital Albert Einstein, São Paulo 05652-900, São Paulo, Brazil; 5 Plataforma Científica Pasteur/USP-SSPU, Universidade de São Paulo, São Paulo 05508-020, São Paulo, Brazil

**Keywords:** COVID-19, dermatological manifestations, MTHFR mutations, Zeta variant

## Abstract

The present case study describes the dermatological manifestations of COVID-19 in a patient with genetic thrombophilia (MTHFR–C677T mutation) and the identification of a SARS-CoV-2 variant of interest (VOI). A female patient, 47 years old, unvaccinated, with thrombophilia, was diagnosed with COVID-19. She presented with urticarial and maculopapular eruptions from the seventh day of symptoms, which progressed to multiple lesions with dark centers (D-dimer value > 1450 ng/mL). The dermatological manifestations disappeared after 30 days, corroborating the reduction in D-dimer levels. Viral genome sequencing revealed infection by the VOI Zeta (P.2). Antibody testing, performed 30 days after the onset of symptoms, detected only IgG. The virus neutralization test showed the highest neutralizing titer for a P.2 strain, validating the genotypic identification. Lesions were suggested to be due to infection in skin cells causing a direct cytopathic effect or release of pro-inflammatory cytokines triggering erythematous and urticarial eruptions. In addition, vascular complications are also proposed to be due to the MTHFR mutation and increased D-dimer values. This case report is an alert about COVID-19 in patients with pre-existing vascular diseases, especially in unvaccinated patients, by VOI.

## 1. Introduction

Respiratory tract manifestations are the main symptoms of coronavirus disease 2019 (COVID-19); however, a wide range of clinical signs and symptoms associated with other systems may occur [1]. Patients’ inflammatory state, such as a hyperinflammatory response, under certain conditions or comorbidities could explain the clinical variables found in COVID-19 [2]. Cutaneous manifestations, for example, appear in a prevalence that varies from 0.2 to 45.6% [3] and can be directly related to the virus or complications of the infection [4]. They can be classified into different types: pseudochills, exanthematous lesions with macules and papules, urticarial, vesicular, or vaso-occlusive forms [5]. The association of these signs is relevant for the clinical diagnosis, as they can assist in early detection or even serve as a prognosis of moderate or severe forms of infection [6]. In addition, it can help in understanding the pathogenesis of the disease and the adoption of infection control policies.

Abnormalities of hemostatic parameters were also readily related to poor prognosis in COVID-19, possibly associated with endothelial injury, stasis, and hypercoagulability in a severe inflammatory state [7]. Here, we describe a dermatological manifestation in an unvaccinated patient, with thrombophilia and mutation in the methylenetetrahydrofolate reductase (MTHFR) enzyme, during COVID-19 caused by a variant of interest (VOI) of severe acute respiratory syndrome coronavirus 2 (SARS-CoV-2).

## 2. Case Presentation

**Figure 1:**
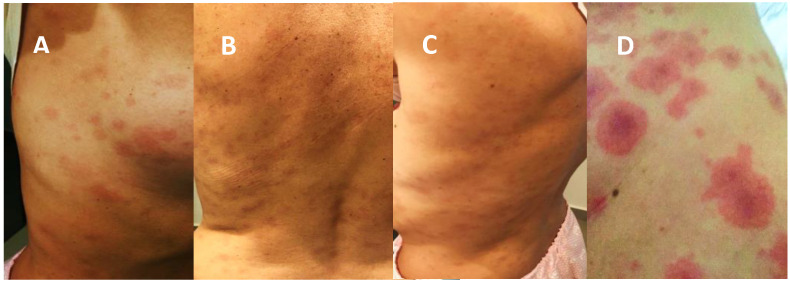
Dermatological manifestations in a patient with COVID-19. Cutaneous lesions started from the seventh day of symptoms in the form of urticaria and maculopapular rashes (**A**–**C**), with evolution to multiple lesions with dusky centers on the ninth day of symptoms (**D**).

A 47-year-old woman sought medical care presenting with 3 days of sore throat. Nasopharyngeal secretion was collected using a rayon swab and maintained in viral transport medium (VTM: DMEM containing penicillin–1000 IU/mL, streptomycin–1000 μg/mL, and amphotericin B–25 μg/mL) to diagnose COVID-19 by real-time reverse transcription polymerase chain reaction (Applied Biosystem, Waltham, MA, USA) [8]. The nasal sample was kept at −80 °C for further characterization. The patient was prescribed therapy, which was to last 5 days, of azithromycin (500 mg, 12/12h), vitamin B12 (0.4 mg), and zinc (29 mg). Three days later, the patient returned to medical care still presenting a sore throat accompanied by fever and tachycardia. Oral dexamethasone (20 mg, 6/6h, for 5 days) was added to the treatment. On the seventh day of infection, the patient reported anosmia, ageusia, and the appearance of urticarial and maculopapular rashes, initially on the limbs and later on the neck and face, spreading to the rest of the body (Figure 1 A–C). On the ninth day of symptoms, she presented multiple lesions with dusky centers (Figure 1D).

**Figure 2:**
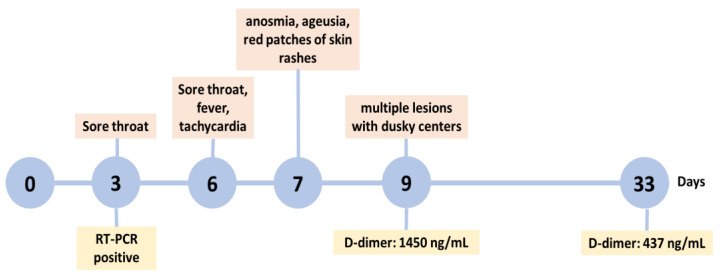
Timeline of the clinical manifestations and laboratory features exhibited by the patient. The numbers (0–33) represent the days on which the symptoms were reported. RT-PCR positive, positive result for COVID-19 by real-time reverse transcription polymerase chain reaction. D-dimer reference value: 198 ng/mL.

The patient sought care from a dermatologist who requested further tests and maintained therapy with vitamin B and zinc, but dexamethasone was replaced by prednisone (20 mg, every 6 h). The imaging test was normal, but D-dimer was elevated: 1450 ng/mL (reference value: 198 ng/mL). D-dimer levels remained high on the following day, and an increase in C-reactive protein–CRP (11.1 mg/mL, reference value < 10.0 mg/mL) was also observed. On this day, the patient informed the doctor that she had genetic thrombophilia with a homozygous mutation of 5-methyltetrahydrofolate reductase (MTHFR) C677T. Treatment with rivaroxaban (20 mg, 1 every 24 h, for 60 days) was immediately started, together with prednisone (20 mg, 1 every 24 h), in addition to the interruption of physical activities. The dermatological manifestations disappeared 30 days after their onset, concomitantly with the decrease in D-dimer to 437 ng/mL. The timeline of clinical manifestations and laboratorial examinations is presented in Figure 2.

Further analysis confirmed the identification of SARS-CoV-2 by genome sequencing. Thus, viral RNA extraction was performed with the QIAamp Viral RNA Mini Kit (Qiagen, Hilden, Germany), followed by DNAse RQI (Promega, Sáo Paulo, Brazil) treatment. Human ribosomal RNA depletion was performed using the QIAseq Fast Select RNA Removal Kit (Qiagen, Hilden, Germany). The viral RNA was subjected to random amplification in two steps, according to Greninger et al. [9], with minor modifications. The sequencing library preparation for the Illumina platform was performed with the Nextera XT Kit (Illumina, San Diego, CA, USA), according to manufacturer’s instructions. Libraries were quantified using the Qubit fluorimetric method (Thermo Fisher Scientific, Waltham, MA, USA) and loaded onto the NextSeq 550 instrument for sequencing with 300 paired-end cycle kits (Illumina, San Diego, CA, USA) at Hospital Israelita Albert Einstein (São Paulo, SP, Brazil). The data were then filtered and trimmed, obtaining a Phred score of >20. The genome was assembled by the “ab initio” strategy with the reference genome NC_045512.2 (SAR-CoV-2), using SPADES software v.3.13.1. The assembly generated a genome of 299757 base pairs in size. The whole-genome sequence of LAVIR-MA-UEL was submitted at DDBJ/ENA/GenBank under the submission number SUB12140327. The data presented here are the first version. After these analyses, it was identified as the variant of interest (VOI) “Zeta” or P.2, named as SARS-CoV-2/human/BRA/LAVIR-MA-UEL/2021 (GenBank SUB12140327), which showed the classic mutations in the spike glycoprotein: E484K, D614G, and V1176F.

Blood samples were also collected within 30 and 60 days of the onset of the patient’s symptoms for serology and virus neutralization tests (VNT). The assay for neutralizing antibodies from serum samples was performed using the cytopathic effect-based virus neutralization test (CPE-VNT) [10]. Briefly, Vero cells (ATCC CCL-81, 10^4^ cells/mL) were cultured before infection (24 h) in a 96-well microplate. Serum samples were inactivated at 56 °C for 30 min. Serial dilutions (8 dilutions, 2-fold) of each serum (1:20 to 1:2560) were used. The separated dilutions were mixed (*v*/*v*) with the TCID50/mL from three different strains of SARS-CoV-2: Wuhan (GenBank MT350282), Zeta, or P.2 (EPI_ISL_770561) and Gamma or P.1 (EPI_ISL_1060981) and pre-incubated for 1 h, at 37 °C, for virus neutralization. The mixture (serum plus virus) was added to the cell monolayer and incubated for 72 h at 37 °C and 5% CO_2_. Virus neutralization titer or VNT100 was considered the highest serum dilution capable of neutralizing viral replication (absence of CPE–cytopathic effect). For each assay, positive and negative controls were used, consisting of strong and safe serum (RT-PCR positive + PRNT90) and pre-outbreak serum. The CPE-VNT experiment was conducted in a biosafety level 3 laboratory [10]. Statistical analyses were obtained through GraphPad Prism (version 5.0). One-way ANOVA followed by Tukey’s test was applied to determine the difference between group means. Results were expressed as the mean ± standard deviation (SD). A *p*-value of <0.001 was considered statistically significant. The experiments were conducted in duplicate.

**Figure 3:**
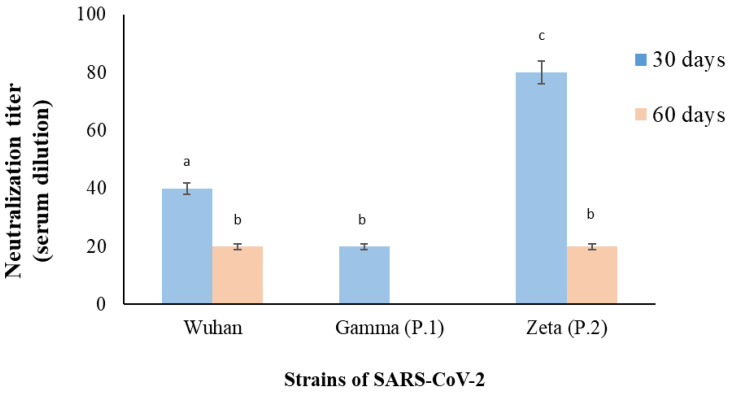
SARS-CoV-2 virus neutralization test (VNT). The serum samples were collected 30 and 60 days after the onset of the patient’s symptoms and the assay and the dilutions were mixed with three different strains of SARS-CoV-2 (Wuhan, Gamma or P.1, Zeta or P.2). The neutralization titer was determined as the highest dilution of serum that neutralized virus growth (absence of cytopathic effect). Results were expressed as the mean ± SD. Different lowercase letters (a, b, c) indicate significant differences among groups (*p* < 0.001).

The antibody testing (IgG and IgM), carried out 30 days after the onset of symptoms, was positive only for IgG (6.95 AU/mL, reference value: reagent > 1.1 AU/mL), reinforcing the hypothesis that remission also occurred through viral neutralization by the patient’s immune system. The VNT result showed the highest neutralizing titer for a P.2 strain, followed by Wuhan, and the lowest for P.1, corroborating the genotypic identification of the Zeta variant of SARS-CoV-2 as the causal agent of the case presented. Interestingly, the 60-day serum weakly neutralized P.2 and Wuhan and was negative for the P.1 strain (Figure 3).

The study was conducted in accordance with the Declaration of Helsinki and approval by the Ethics Committee in Research involving Human Beings of the State University of Londrina–CEP/UEL and the National Research Ethics Committee (CEPE), guidance (CAAE: 32492720.9.0000.5231)

## 3. Discussion

This case describes an unvaccinated patient with COVID-19 initially presenting with urticarial eruptions, followed by erythematous lesions containing macules and papules, predominantly involving the trunk (Figure 1). Cutaneous manifestations may occur at different times of the disease and may indicate whether the lesions are induced by the virus or by the host immune response to the infection. In virus-induced lesions, it is suggested that the virus binds to angiotensin-converting enzyme 2 (ACE2) receptors expressed on skin keratinocytes, causing a direct CPE [11,12]. Tan et al. [5] suggest that cutaneous manifestations of COVID-19 present demographic differences in prevalence, being more frequent in middle-aged (41.9 years) European and American female patients, who display a high survival rate. Urticarial and maculopapular lesions exhibit similar patterns, co-occurring with other symptoms such as anosmia/ageusia (23%) and are usually of short duration (6 to 8 days). Pruritus is most common in urticarial lesions (92%), followed by maculopapular lesions (56%) [13,14].

Cutaneous manifestations may vary according to viral cutaneous tropism or the induction of immune response and may be related to specific variants of SARS-CoV-2 [12]. However, information on cutaneous manifestations associated with SARS-CoV-2 variants is very limited [15] owing to the reduced number of case reports and few dermatological studies with viral identification by sequencing, especially in regions with the co-circulation of several strains. In the present study, the SARS-CoV-2/human/BRA/LAVIR-MA-UEL/2021 was identified as the variant of interest (VOI) “Zeta” or P.2 and presented the classical mutational signatures in the spike (S) glycoprotein: E484K, D614G, and V1176F. These mutations have already been reported in several variants, and the E484K substitution is well known for its relationship with immune evasion [16]. A study by Yadav et al. [17] showed that P.2-infected Syrian hamsters showed a marked loss in body weight and viral replication in the respiratory tract with severe pneumonia. These results corroborate cases of reinfection by strains with mutations in the spike glycoprotein, such as E484K in the receptor binding domain (RBD) [16,18].

These mutations could also explain the absence of neutralization observed for the P.1 variant in the VNT. Souza et al. [19] proposed an 8.6-fold reduction in the neutralization capacity of the P.1 strain in plasma from individuals previously infected with SARS-CoV-2. Studies suggest that the longer duration of neutralizing antibodies to SARS-CoV-2 is associated with the severity of the infection, being lower for patients with mild and asymptomatic conditions [20]. Furthermore, the neutralizing activity of antibodies in plasma appears to decrease significantly between 1.3 and 6.2 months after infection [21]. Nevertheless, a decrease in antibody levels does not necessarily equate to a loss of immunity. Even at undetectable levels, memory B and T cells can generate a faster or more effective response to future reinfections. The local synthesis of mucosal antibodies in the airways can also help prevent or hinder infection [22].

The P.2 variant was described for the first time in Rio de Janeiro, Brazil, and possibly emerged in July 2020 [23]. P.2 derived from the B.1.1.28 lineage, the same lineage that gave rise to the variant of concern (VOC) P.1 or Gamma. D614G and V1176F mutations had already been demonstrated in B.1.1.28 [24] and are positively correlated with increased mortality rates. D614G is associated with higher infectivity and higher viral load [25], in addition to increasing the prevalence of anosmia in COVID-19 patients [26], a symptom also reported by the patient in the present study on the seventh day of infection. V1176F is related to a more significant interaction with ACE2 due to the stabilization of the spike protein trimeric complex [27]. Despite few studies reporting the association between SARS-CoV-2 variants and dermatological manifestations, a reduction in rash cases was reported during the circulation period of Delta (17.6%) and Omicron (11.4%) variants, but with a longer duration. For the Delta variant, the occurrence of gangrene was also suggested, in addition to acral and livedoid lesions, possibly related to the involvement of the vascular system caused by this variant [28]. Thus, cutaneous manifestations can be predictive of infection by SARS-CoV-2 and their monitoring could infer the identification of new variants [29]. More studies like this are needed to establish this relationship.

Regarding the immune response to COVID-19 infection, several mechanisms have been proposed. The possible mechanisms that generate urticarial lesions involve direct degranulation of mast cells [30] or those induced by nonspecific drugs supposedly used as early treatment [31,32,33]. In this case, during the anamnesis, the patient denied taking such drugs. The cutaneous manifestations can also be explained by a possible endotheliitis evidenced by the intracellular detection of the virus in histopathological studies of patients with COVID-19 [34]. The binding of SARS-CoV-2 glycoproteins to the ACE2 receptor, responsible for the degradation of vasodilating kinins such as bradykinin, can also result in increased vascular permeability, accentuating edematous or urticarial lesions [35]. Furthermore, the activation of bradykinin receptors can trigger a pro-inflammatory effect, contributing to inflammatory skin manifestations [36,37]. The release of pro-inflammatory cytokines, often called a cytokine storm, can reach the skin and stimulate dermal inflammatory cells, triggering erythematous and urticarial eruptions [38]. It is estimated that chemokine response and inflammatory cell infiltration occur seven to ten days after symptom onset, leading to an increased risk of vascular hyperpermeability and irreversible complications [39].

The case described here also highlights the elevation of serum D-dimer value, mentioned by several studies as the most common clotting abnormality in severe COVID-19 and an independent risk factor for death, especially when values are greater than 1000 ng/mL [40]. The elevation of this fibrin degradation product may be associated with disseminated intravascular coagulation, in which there is a reduction in clotting factors and, consequently, in the occurrence of thrombosis of small and medium vessels. In parallel with consumption, there is frequent destruction of fibrin networks, increasing circulating D-dimer values [41]. Necrotic skin lesions may occur in 6% of patients with cutaneous disease related to COVID-19 [13]. Another altered clinical parameter was serum CRP, which was also suggested as an early marker of infection, inflammation, and severity of COVID-19. CRP binds preferentially to phosphocholine expressed on the surface of damaged cells, activating the classical complement pathway and modulating phagocytic activity to eliminate damaged cells from the body. In this way, when inflammation or tissue damage is resolved, the concentration of CRP decreases. The association between elevated CRP concentration and worsening of patients with COVID-19 is proposed for values of 26.9 mg/L [42]. However, in the case presented, the elevation was discrete (11.1 mg/L).

In our patient, the cutaneous manifestation evolution can also be explained by the presence of genetic thrombophilia as a risk factor. In suspected COVID-19-associated coagulopathies, screening for thrombophilia is suggested [43]. The MTHFR–C677T mutation and its influence on the immune status, as well as risk factors with thromboembolic phenomena, have already been correlated in severe COVID [44,45,46]. However, the relationship with cutaneous manifestations associated with SARS-CoV-2 VOI is rare. MTHFR is an enzyme that regulates fundamental processes in cellular physiology. The C677T mutation in the MTHFR encoding gene, in which cytosine is replaced by thymine at position 677, has been suggested as the most common genetic cause of hyperhomocysteinemia (H-Hcy) [47]. Some studies [48,49,50] have demonstrated an association between H-Hcy and peripheral vascular, cerebrovascular, and coronary artery disease. Acute H-Hcy activates a pro-inflammatory cascade through the upregulation of nuclear transcription factor (NF-kB) in neutrophils and macrophages, which release a large amount of reactive oxygen species (ROS), potentiating oxidative stress. The increased production of ROS, associated with acute respiratory viral infection by SARS, additionally overloads the oxidative defense system. Moreover, ROS-activated NF-kB can accelerate viral replication, as previously demonstrated in SARS-CoV-1 infection [51].

Thus, some mechanisms can be suggested for the MTHFR mutation, with high levels of the prothrombotic factor H-Hcy and the development of skin lesions due to coagulation disorders, characteristic of severe COVID-19: increased pro-inflammatory cytokines (IL-6, IL-2, IL-4, TNFα) or “cytokine storm”, platelet cell hyperactivity, platelet aggregation, clot formation, and thrombus development. In addition to endothelial dysfunction, promoted by limited nitric oxide generation, and endothelial disruption, combined with irreversible thrombomodulin inactivation, by ROS [52,53,54,55]. The presence of the MTHFR mutation can, therefore, make the disease related to the coagulation cascade more complex, with a greater possibility of death due to thrombosis [56].

The intake of vitamins, including vitamin B and zinc, is suggested as a supportive nutritional intervention in active COVID-19, contributing to the antiviral effect [57]. In the present case study, the nutritional intervention was performed immediately after confirmed diagnosis of COVID-19, which may have positively influenced the outcome of the infection. The treatment of cutaneous manifestations includes low-dose systemic corticosteroid therapy, which may be combined with antihistamines [58]. In the case presented, the urticarial lesion disappeared within 24 h, followed by a maculopapular lesion, lasting about 30 days, with constant use of corticosteroids (prednisone) for remission.

The present study reports the first Brazilian case of cutaneous manifestations in COVID-19, due to infection by VOI Zeta, associated with genetic thrombophilia caused by MTHFR mutation. Thrombophilia and hematological disorders are among the leading causes of death in COVID-19 [41,59,60], and a strong correlation with the MTHFR C677T gene polymorphism has been proposed. Data stratified into ethnic groups, regarding the frequency of MTHFR C677T and the incidence/mortality of COVID-19, show its worldwide distribution, with increasing frequency from South to North and East to West, especially in the Latin American population, which accounted for approximately 50% of frequency [61]. In the Brazilian population, the frequency of MTHFR C677T seems to be heterogeneous due to miscegenation; however, some local studies suggest a variation from 37.3% to 58.5% [62,63,64,65,66]. In addition, the study associates the underlying disease (thrombophilia) with the occurrence of dermatological manifestations in COVID-19. Although systematic reviews suggest about 6% of this occurrence, the real frequency remains uncertain, ranging from 0.2% to 45% [3]. It is noteworthy that many of these results were obtained from electronic medical records, remote assessments carried out by patients, or even through the inclusion of suspected (unconfirmed) cases, with no exclusion of other dermatoses. The study performed by Dupont et al. [67] analyzed the frequency of cutaneous manifestations in two groups: electronic medical records and face-to-face consultations with dermatologists, resulting in frequencies of 1.27% and 10.56%, respectively. Underreporting contributes to a decrease in the frequency of real cases, and cannot be ruled out. Here, the onset and evolution of cutaneous manifestations, which occurred during SARS-CoV-2 infection, were followed in person and through clinical and laboratory parameters, including genotyping and association with the monitoring of D-dimer levels, strongly correlating with thrombophilia and excluding the occurrence of other viral infections as well as adverse drug reactions. It is important to highlight that the present case involved a patient who had not been vaccinated against COVID-19, reinforcing the importance of vaccination, which is proven to reduce the onset of serious illnesses and death [68]. Therefore, this study is inherently important for the understanding of the pathogenesis of COVID-19, as it corroborates the appearance of cutaneous manifestations, associated with a VOI with classical mutational signatures, in a state of hypercoagulability (thrombophilia). Despite the limitation involving a single patient, viral, genetic, and environmental conditions seem to be common to the study region, alerting the scientific and health professional communities.

## 4. Conclusions

The present case study described the occurrence of dermatological manifestation during VOI infection in COVID-19 in a patient with genetic thrombophilia due to MTHFR mutation. Clinical manifestations may be related to viral strain and host factors. We suggest that cutaneous manifestations can be predictive of SARS-CoV-2 infection and their monitoring can contribute to the identification of new variants. More studies on this subject are needed to establish this relationship.

## Data Availability

All data obtained in the present study were demonstrated in the manuscript. A copy of the data is available for review by the Editor of this journal.

## References

[1] Thakur V., Ratho R.K., Kumar P., Bhatia S.K., Bora I., Mohi G.K., Saxena S.K., Devi M., Yadav D., Mehariya S. (2021). Multi-Organ Involvement in COVID-19: Beyond Pulmonary Manifestations. J. Clin. Med..

[2] Kreutmair S., Kauffmann M., Unger S., Ingelfinger F., Núñez N.G., Alberti C., De Feo D., Krishnarajah S., Friebel E., Ulutekin C. (2022). Preexisting comorbidities shape the immune response associated with severe COVID-19. J. Allergy Clin. Immunol..

[3] Seque C.A., Enokihara M.M.S.e.S., Porro A.M., Tomimori J. (2022). Skin Manifestations Associated with COVID-19. An. Bras. Dermatol..

[4] Suchonwanit P., Leerunyakul K., Kositkuljorn C. (2020). Cutaneous Manifestations In COVID-19: Lessons Learned From Current Evidence. J. Am. Acad. Dermatol..

[5] Tan S.W., Tam Y.C., Oh C.C. (2021). Skin Manifestations of COVID-19: A Worldwide Review. JAAD Int..

[6] Genovese G., Moltrasio C., Berti E., Marzano A.V. (2021). Skin Manifestations Associated with COVID-19: Current Knowledge and Future Perspectives. Dermatology.

[7] Bilaloglu S., Aphinyanaphongs Y., Jones S., Iturrate E., Hochman J., Berger J.S. (2020). Thrombosis in Hospitalized Patients with COVID-19 in a New York City Health System. JAMA J. Am. Med. Assoc..

[8] Corman V.M., Landt O., Kaiser M., Molenkamp R., Meijer A., Chu D.K.W., Bleicker T., Brünink S., Schneider J., Schmidt M.L. (2020). Detection of 2019 Novel Coronavirus (2019-NCoV) by Real-Time RT-PCR. Eurosurveillance.

[9] Greninger A.L., Naccache S.N., Federman S., Yu G., Mbala P., Bres V., Stryke D., Bouquet J., Somasekar S., Linnen J.M. (2015). Rapid Metagenomic Identification of Viral Pathogens in Clinical Samples by Real-Time Nanopore Sequencing Analysis. Genome Med..

[10] Wendel S., Kutner J.M., Machado R., Fontão-Wendel R., Bub C., Fachini R., Yokoyama A., Candelaria G., Sakashita A., Achkar R. (2020). Screening for SARS-CoV-2 Antibodies in Convalescent Plasma in Brazil: Preliminary Lessons from a Voluntary Convalescent Donor Program. Transfusion.

[11] Agnihothri R., Fox L.P. (2021). Clinical Patterns and Morphology of COVID-19 Dermatology. Dermatol. Clin..

[12] Burlando M., Russo R., Cozzani E., Parodi A. (2021). COVID-19 “Second Wave” and Vaccines: The Dermatologists’ Perspective. Int. J. Dermatol..

[13] Galván Casas C., Català A., Carretero Hernández G., Rodríguez-Jiménez P., Fernández-Nieto D., Rodríguez-Villa Lario A., Navarro Fernández I., Ruiz-Villaverde R., Falkenhain-López D., Llamas Velasco M. (2020). Classification of the Cutaneous Manifestations of COVID-19: A Rapid Prospective Nationwide Consensus Study in Spain with 375 Cases. Br. J. Dermatol..

[14] Català A., Galván-Casas C., Carretero-Hernández G., Rodríguez-Jiménez P., Fernández-Nieto D., Rodríguez-Villa A., Navarro-Fernández Í., Ruiz-Villaverde R., Falkenhain-López D., Llamas-Velasco M. (2020). Maculopapular Eruptions Associated to COVID-19: A Subanalysis of the COVID-Piel Study. Dermatol. Ther..

[15] Sławińska M., Nowicki R.J. (2020). Dermatological Manifestations of COVID-19: A Practical Summary of the Current State of Knowledge. Przegl. Dermatol..

[16] Nonaka C.K.V., Franco M.M., Gräf T., de Lorenzo Barcia C.A., de Ávila Mendonça R.N., de Sousa K.A.F., Costa Neiva L.M., Fosenca V., Mendes A.V.A., de Aguiar R.S. (2021). Genomic Evidence of SARS-CoV-2 Reinfection Involving E484K Spike Mutation, Brazil. Emerg. Infect. Dis..

[17] Yadav P., Mohandas S., Sarkale P., Nyayanit D., Shete A., Sahay R., Potdar V., Baradkar S., Gupta N., Sapkal G. (2022). Isolation of SARS-CoV-2 B.1.1.28.2 (P2) Variant and Pathogenicity Comparison with D614G Variant in Hamster Model. J. Infect. Public Health.

[18] Garcia-Beltran W.F., Lam E.C., St. Denis K., Nitido A.D., Garcia Z.H., Hauser B.M., Feldman J., Pavlovic M.N., Gregory D.J., Poznansky M.C. (2021). Multiple SARS-CoV-2 Variants Escape Neutralization by Vaccine-Induced Humoral Immunity. Cell.

[19] Souza W.M., Amorim M.R., Sesti-Costa R., Coimbra L.D., Brunetti N.S., Toledo-Teixeira D.A., de Souza G.F., Muraro S.P., Parise P.L., Barbosa P.P. (2021). Neutralisation of SARS-CoV-2 Lineage P.1 by Antibodies Elicited through Natural SARS-CoV-2 Infection or Vaccination with an Inactivated SARS-CoV-2 Vaccine: An Immunological Study. Lancet Microbe.

[20] Chia W.N., Zhu F., Ong S.W.X., Young B.E., Fong S.W., le Bert N., Tan C.W., Tiu C., Zhang J., Tan S.Y. (2021). Dynamics of SARS-CoV-2 Neutralising Antibody Responses and Duration of Immunity: A Longitudinal Study. Lancet Microbe.

[21] Pang N.Y.L., Pang A.S.R., Chow V.T., Wang D.Y. (2021). Understanding Neutralising Antibodies against SARS-CoV-2 and Their Implications in Clinical Practice. Mil. Med. Res..

[22] Röltgen K., Powell A.E., Wirz O.F., Stevens B.A., Hogan C.A., Najeeb J., Hunter M., Wang H., Sahoo M.K., Huang C.H. (2020). Defining the Features and Duration of Antibody Responses to SARS-CoV-2 Infection Associated with Disease Severity and Outcome. Sci. Immunol..

[23] Voloch C.M., da Silva Francisco R., de Almeida L.G.P., Cardoso C.C., Brustolini O.J., Gerber A.L., Guimarães A.P.d.C., Mariani D., da Costa R.M., Ferreira O.C. (2021). Genomic Characterization of a Novel SARS-CoV-2 Lineage from Rio de Janeiro, Brazil. J. Virol..

[24] Demoliner M., da Silva M.S., Gularte J.S., Hansen A.W., de Almeida P.R., Weber M.N., Heldt F.H., Silveira F., Filippi M., de Abreu Góes Pereira V.M. (2021). Predominance of SARS-CoV-2 P.1 (Gamma) Lineage Inducing the Recent COVID-19 Wave in Southern Brazil and the Finding of an Additional S: D614A Mutation. Infect. Genet. Evol..

[25] Korber B., Fischer W.M., Gnanakaran S., Yoon H., Theiler J., Abfalterer W., Hengartner N., Giorgi E.E., Bhattacharya T., Foley B. (2020). Tracking Changes in SARS-CoV-2 Spike: Evidence That D614G Increases Infectivity of the COVID-19 Virus. Cell.

[26] von Bartheld C.S., Hagen M.M., Butowt R. (2021). The D614G Virus Mutation Enhances Anosmia in COVID-19 Patients: Evidence from a Systematic Review and Meta-Analysis of Studies from South Asia. ACS Chem. Neurosci..

[27] Majumdar P., Niyogi S. (2021). SARS-CoV-2 Mutations: The Biological Trackway towards Viral Fitness. Epidemiol. Infect..

[28] Luo R., Delaunay-Moisan A., Timmis K., Danchin A. (2021). SARS-CoV-2 Biology and Variants: Anticipation of Viral Evolution and What Needs to Be Done. Environ. Microbiol..

[29] Visconti A., Murray B., Rossi N., Wolf J., Ourselin S., Spector T.D., Freeman E.E., Bataille V., Falchi M. (2022). Cutaneous Manifestations of SARS-CoV-2 Infection during the Delta and Omicron Waves in 348,691 UK Users of the UK ZOE COVID Study App. Br. J. Dermatol..

[30] Lai C.C., Ko W.C., Lee P.I., Jean S.S., Hsueh P.R. (2020). Extra-Respiratory Manifestations of COVID-19. Int. J. Antimicrob. Agents.

[31] Singh H., Kaur H., Singh K., Sen C.K. (2021). Cutaneous Manifestations of COVID-19: A Systematic Review. Adv. Wound Care.

[32] Jindal R., Chauhan P. (2020). Cutaneous Manifestations of Coronavirus Disease 2019 in 458 Confirmed Cases: A Systematic Review. J. Fam. Med. Prim. Care.

[33] Martinez-Lopez A., Cuenca-Barrales C., Montero-Vilchez T., Molina-Leyva A., Arias-Santiago S. (2020). Review of Adverse Cutaneous Reactions of Pharmacologic Interventions for COVID-19: A Guide for the Dermatologist. J. Am. Acad. Dermatol..

[34] Florêncio F.K.Z., de Oliveira Tenório M., Andrade A.R.M., de Lima S.G. (2020). Angioedema, Endothelium, ACE2, and Bradykinin—Interrelationships in COVID-19: A Case Report. Medicina.

[35] Kroumpouzos G. (2021). Cutaneous Manifestations of COVID-19: An Unusual Presentation with Edematous Plaques and Pruritic, Erythematous Papules, and Comment on the Role of Bradykinin Storm and Its Therapeutic Implications. Dermatol. Ther..

[36] Ehrenfeld P., Millan C., Matus C.E., Figueroa J.E., Burgos R.A., Nualart F., Bhoola K.D., Figueroa C.D. (2006). Activation of Kinin B1 Receptors Induces Chemotaxis of Human Neutrophils. J. Leukoc. Biol..

[37] Kenne E., Rasmuson J., Renné T., Vieira M.L., Müller-Esterl W., Herwald H., Lindbom L. (2019). Neutrophils Engage the Kallikrein-Kinin System to Open up the Endothelial Barrier in Acute Inflammation. FASEB J..

[38] Kaya G., Kaya A., Saurat J.-H. (2020). Clinical and Histopathological Features and Potential Pathological Mechanisms of Skin Lesions in COVID-19: Review of the Literature. Dermatopathology.

[39] Salimi-Jeda A., Abbassi S., Mousavizadeh A., Esghaie M., Bokharaei-Salim F., Jeddi F., Shafaati M., Abdoli A. (2021). SARS-CoV-2: Current Trends in Emerging Variants, Pathogenesis, Immune Responses, Potential Therapeutic, and Vaccine Development Strategies. Int. Immunopharmacol..

[40] Chen N., Zhou M., Dong X., Qu J., Gong F., Han Y., Qiu Y., Wang J., Liu Y., Wei Y. (2020). Epidemiological and Clinical Characteristics of 99 Cases of 2019 Novel Coronavirus Pneumonia in Wuhan, China: A Descriptive Study. Lancet.

[41] Miesbach W., Makris M. (2020). COVID-19: Coagulopathy, Risk of Thrombosis, and the Rationale for Anticoagulation. Clin. Appl. Thromb. Hemost..

[42] Ali N. (2020). Elevated Level of C-Reactive Protein May Be an Early Marker to Predict Risk for Severity of COVID-19. J. Med. Virol..

[43] Capoferri G., Daikeler T., Mühleisen B., Trendelenburg M., Müller S. (2022). Cutaneous Leukocytoclastic Vasculitis Secondary to COVID-19 Infection Leading to Extensive Skin Necrosis. Clin. Dermatol..

[44] Karst M., Hollenhorst J., Achenbach J. (2020). Life-Threatening Course in Coronavirus Disease 2019 (COVID-19): Is There a Link to Methylenetetrahydrofolic Acid Reductase (MTHFR) Polymorphism and Hyperhomocysteinemia?. Med. Hypotheses.

[45] Annunziata A., Coppola A., di Spirito V., Cauteruccio R., Marotta A., di Micco P., Fiorentino G. (2021). The Angiotensin Converting Enzyme Deletion/Deletion Genotype Is a Risk Factor for Severe COVID-19: Implication and Utility for Patients Admitted to Emergency Department. Medicina.

[46] Cantanhede M.H.D., Sarges K.M.d.L., Leite M.d.M., Miyajima F., dos Santos E.J.M. (2022). Suscetibilidade de polimorfismos genéticos a trombofilia e seu papel na COVID-19. Braz. J. Infect. Dis..

[47] Yafei W., Lijun P., Jinfeng W., Xiaoying Z. (2012). Is the Prevalence of MTHFR C677T Polymorphism Associated with Ultraviolet Radiation in Eurasia?. J. Hum. Genet..

[48] Yadav U., Kumar P., Gupta S., Rai V. (2017). Distribution of MTHFR C677T Gene Polymorphism in Healthy North Indian Population and an Updated Meta-Analysis. Indian J. Clin. Biochem..

[49] Wilcken B., Bamforth F., Li Z., Zhu H., Ritvanen A., Redlund M., Stoll C., Alembik Y., Dott B., Czeizel A.E. (2003). Geographical and Ethnic Variation of the 677C>T Alleleof 5,10 Methylenetetrahydrofolate Reductase (MTHFR): Findings from over 7000 Newborns from 16 Areas Worldwide. J. Med. Genet..

[50] Russo G.T., Friso S., Jacques P.F., Rogers G., Cucinotta D., Wilson P.W.F., Ordovas J.M., Rosenberg I.H., Selhub J. (2003). Age and Gender Affect the Relation between Methylenetetrahydrofolate Reductase C677T Genotype and Fasting Plasma Homocysteine Concentrations in the Framingham Offspring Study Cohort. J. Nutr..

[51] Smits S.L., de Lang A., van den Brand J.M.A., Leijten L.M., van Ijcken W.F., Eijkemans M.J.C., van Amerongen G., Kuiken T., Andeweg A.C., Osterhaus A.D.M.E. (2010). Exacerbated Innate Host Response to SARS-CoV in Aged Non-Human Primates. PLoS Pathog..

[52] Berbert A. (2021). Further comment on articles pertaining to: “Homocysteine as a potential predictor of cardiovascular risk in patients with COVID-19”. Med. Hypotheses.

[53] Lord N., Ruwart M.J. (2020). Homocysteine and the SARS-CoV-2 Coronavirus—The X factor of severe disease and death. SSRN.

[54] Ponti G., Roli L., Oliva G., Manfredini M., Trenti T., Kaleci S., Iannella R., Balzano B., Coppola A., Fiorentino G. (2021). Homocysteine (Hcy) assessment to predict outcomes of hospitalized COVID-19 patients: A multicenter study on 313 COVID-19 patients. Clin. Chem. Lab. Med..

[55] Tavares L.S., Ortiz J.V. (2021). Development of thrombosis in patients with and without SARS-Cov-2 infection—Literature review. Res. Soc. Dev..

[56] de la Morena-Barrio M.E., Bravo-Pérez C., de la Morena-Barrio B., Orlando C., Cifuentes R., Padilla J., Miñano A., Herrero S., Marcellini S., Revilla N. (2021). A pilot study on the impact of congenital thrombophilia in COVID-19. Eur. J. Clin. Investig..

[57] Zhang L., Liu Y. (2020). Potential Interventions for Novel Coronavirus in China: A Systematic Review. J. Med Virol..

[58] Fernández-Lázaro D., Garrosa M. (2021). Identification, Mechanism, and Treatment of Skin Lesions in COVID-19: A Review. Viruses.

[59] Iba T., Levy J.H., Levi M., Thachil J. (2020). Coagulopathy in COVID-19. J. Thromb. Haemost..

[60] Chan N.C., Weitz J.I. (2020). COVID-19 coagulopathy, thrombosis, and bleeding. Blood.

[61] Ponti G., Pastorino L., Manfredini M., Ozben T., Oliva G., Kaleci S., Lannella R., Tomasi A. (2021). COVID-19 spreading across world correlates with C677T allele of the methylenetetrahydrofolate reductase (MTHFR) gene prevalence. J. Clin. Lab. Anal..

[62] Dusse L.M., Carvalho M., Braganca W.F., Paiva S.G., Godoi L.C., Guimaraes D.A., Godoi L.C., Guimarães D.A.M., Fernandes A.P. (2007). Inherited thrombophilias and pre-eclampsia in Brazilian women. Eur. J. Obstet. Gynecol. Reprod. Biol..

[63] Filho I.L., Leite A.C., Moura P.G., Ribeiro G.S., Cavalcante A.C., Azevedo F.C., Andrada-Serpa M.J. (2011). Genetic polymorphisms and cerebrovascular disease in children with sickle cell anemia from Rio de Janeiro, Brazil. Arq. Neuropsiquiatr..

[64] Sabino A.P., Guimarães D.A.M., Ribeiro D.D., Paiva S.G., Dusse L.M., Carvalho M.G., Fernandes A.P. (2007). Increased Factor V Leiden frequency is associated with venous thrombotic events among young Brazilian patients. J. Thromb. Thrombolysis.

[65] Dick-Guareschi J., Fontana J.C., Sanseverino M.T.V., Kubaski F., Sekine L., Mesquita N.F., Onsten T.G.H., Leistner-Segala S. (2022). Prevalence of thrombophilia-associated genetic risk factors in blood donors of a regional hospital in southern Brazil. Hematol. Transfus. Cell Ther..

[66] Soligo A.G., Barini R., Annichino-Bizzacchi J.M. (2017). Prevalence of the MTHFR C677T mutation in fertile and infertile women. Rev. Bras. Ginecol. Obstet..

[67] Dupont L., Duquia R.P., Pizutti G.W., Nunes F.B., Branchini G., Mosquera E.S.B., Bonamigo R.R. (2022). Cutaneous manifestations in patients with COVID-19 treated at an university hospital in southern Brazil. Cureus.

[68] Passarelli-Araujo H., Pott-Junior H., Susuki A.M., Olak A.S., Pescim R.R., Tomimatsu M.F.A.I., Volce C.J., Neves M.A.Z., Silva F.F., Narciso S.G. (2022). The impact of COVID-19 vaccination on case fatality rates in a city in Southern Brazil. Am. J. Infect. Control..

